# *trans*-Tetra­aqua­bis­(isonicotinamide-κ*N* ^1^)nickel(II) bis­(3-hy­droxy­benzoate) tetra­hydrate

**DOI:** 10.1107/S1600536812002218

**Published:** 2012-01-21

**Authors:** Ibrahim Göker Zaman, Nagihan Çaylak Delibaş, Hacali Necefoğlu, Tuncer Hökelek

**Affiliations:** a Department of Chemistry, Kafkas University, 36100 Kars, Turkey; b Department of Physics, Sakarya University, 54187 Esentepe, Sakarya, Turkey; c Department of Physics, Hacettepe University, 06800 Beytepe, Ankara, Turkey

## Abstract

The asymmetric unit of the title compound, [Ni(C_6_H_6_N_2_O)_2_(H_2_O)_4_](C_7_H_5_O_3_)_2_·4H_2_O, contains one-half of the complex cation with the Ni^II^ ion located on an inversion center, a 3-hy­droxy­benzoate counter-anion and two uncoordinated water mol­ecules. Four water O atoms in the equatorial plane around the Ni^II^ ion [Ni—O = 2.052 (2) and 2.079 (2) Å] form a slightly distorted square-planar arrangement, which is completed up to a distorted octa­hedron by the two N atoms [Ni—N = 2.075 (3) Å] from two isonicotinamide ligands. In the anion, the carboxyl­ate group is twisted from the attached benzene ring by 8.8 (3)°. In the crystal, a three-dimensional hydrogen-bonding network, formed by classical O—H⋯O and N—H⋯O hydrogen bonds, consolidates the crystal packing, which also exhibits π–π inter­actions between the benzene and pyridine rings, with centroid–centroid distances of 3.455 (2) and 3.621 (2) Å, respectively.

## Related literature

For general background, see: Bigoli *et al.* (1972 ▶); Krishnamachari (1974 ▶). For related structures, see: Hökelek *et al.* (2009*a* ▶,*b* ▶,*c* ▶,*d* ▶,*e* ▶); Sertçelik *et al.* (2009*a* ▶,*b* ▶).
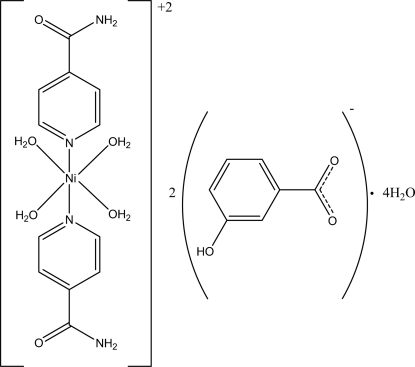


## Experimental

### 

#### Crystal data

[Ni(C_6_H_6_N_2_O)_2_(H_2_O)_4_](C_7_H_5_O_3_)_2_·4H_2_O
*M* *_r_* = 721.29
Monoclinic, 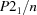

*a* = 6.6884 (3) Å
*b* = 16.9271 (5) Å
*c* = 13.5543 (4) Å
β = 100.186 (3)°
*V* = 1510.37 (9) Å^3^
*Z* = 2
Mo *K*α radiation
μ = 0.73 mm^−1^
*T* = 100 K
0.46 × 0.33 × 0.18 mm

#### Data collection

Bruker Kappa APEXII CCD area-detector diffractometer
Absorption correction: multi-scan (*SADABS*; Bruker, 2005 ▶) *T*_min_ = 0.750, *T*_max_ = 0.877
13495 measured reflections
3723 independent reflections
3365 reflections with *I* > 2σ(*I*)
*R*_int_ = 0.029

#### Refinement

*R*[*F* ^2^ > 2σ(*F* ^2^)] = 0.051
*wR*(*F* ^2^) = 0.157
*S* = 1.26
3723 reflections
256 parameters
12 restraints
H atoms treated by a mixture of independent and constrained refinement
Δρ_max_ = 0.86 e Å^−3^
Δρ_min_ = −0.69 e Å^−3^

### 

Data collection: *APEX2* (Bruker, 2007 ▶); cell refinement: *SAINT* (Bruker, 2007 ▶); data reduction: *SAINT*; program(s) used to solve structure: *SHELXS97* (Sheldrick, 2008 ▶); program(s) used to refine structure: *SHELXL97* (Sheldrick, 2008 ▶); molecular graphics: *ORTEP-3 for Windows* (Farrugia, 1997 ▶); software used to prepare material for publication: *WinGX* (Farrugia, 1999 ▶) and *PLATON* (Spek, 2009 ▶).

## Supplementary Material

Crystal structure: contains datablock(s) I, global. DOI: 10.1107/S1600536812002218/cv5237sup1.cif

Structure factors: contains datablock(s) I. DOI: 10.1107/S1600536812002218/cv5237Isup2.hkl

Additional supplementary materials:  crystallographic information; 3D view; checkCIF report

## Figures and Tables

**Table 1:** Hydrogen-bond geometry (Å, °)

*D*—H⋯*A*	*D*—H	H⋯*A*	*D*⋯*A*	*D*—H⋯*A*
N2—H21⋯O1^i^	0.83 (5)	2.20 (5)	3.018 (4)	169 (4)
N2—H22⋯O8^ii^	0.84 (5)	2.21 (5)	3.012 (4)	159 (5)
O3—H31⋯O7	0.79 (5)	1.91 (5)	2.696 (4)	175 (5)
O5—H51⋯O3^ii^	0.85 (4)	1.87 (4)	2.716 (3)	177 (5)
O5—H52⋯O1^iii^	0.84 (5)	1.99 (6)	2.795 (4)	160 (6)
O6—H61⋯O4^ii^	0.85 (3)	1.86 (3)	2.693 (3)	169 (4)
O6—H62⋯O1^iv^	0.85 (5)	1.87 (5)	2.685 (4)	159 (5)
O7—H71⋯O7^v^	0.78 (4)	2.03 (3)	2.795 (4)	167 (6)
O7—H72⋯O2^vi^	0.85 (5)	1.88 (5)	2.731 (4)	177 (5)
O8—H81⋯O7^vii^	0.77 (4)	2.10 (4)	2.802 (4)	152 (6)
O8—H82⋯O2	0.83 (4)	1.93 (5)	2.752 (4)	171 (4)

Symmetry codes: (i) 
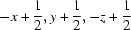
; (ii) 
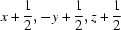
; (iii) 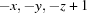; (iv) 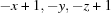; (v) 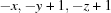; (vi) 
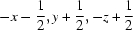
; (vii) 
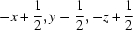
.

**Fig. 1.**
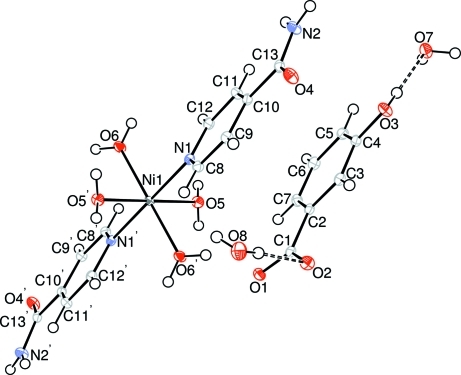
The molecular structure of (I) with the atom-numbering scheme [symmetry code: (') - x, - y, -z]. Displacement ellipsoids are drawn at the 50% probability level. Hydrogen bonds are shown as dashed lines.

## supplementary crystallographic information

### Comment

As a part of our ongoing investigation on transition metal complexes of nicotinamide (NA), one form of niacin (Krishnamachari, 1974), and/or the nicotinic acid derivative *N*,*N*-diethylnicotinamide (DENA), an important respiratory stimulant (Bigoli *et al.*, 1972), the title compound (I) was synthesized and its crystal structure is reported herein.

The asymmetric unit of (I) (Fig. 1) contains one Ni^II^ ion on a centre of symmetry, one isonicotinamide (INA) ligand, one 3-hydroxybenzoate (HB) molecule, two coordinated and two uncoordinated water molecules, respectively. The structures of some DENA and/or NA complexes of Ni^II^ and Co(II) ions, [Ni(C_8_H_5_O_3_)_2_(C_10_H_14_N_2_O)_2_(H_2_O)_2_] (Sertçelik *et al.*, 2009*a*), [Ni(C_6_H_6_N_2_O)_2_(H_2_O)_4_](C_7_H_4_FO_2_)_2_ (Hökelek *et al.*, 2009*a*), [Ni(C_6_H_6_N_2_O)_2_(H_2_O)_4_](C_8_H_5_O_3_)_2_.2(H_2_O) (Hökelek *et al.*, 2009*b*), [Ni(C_7_H_4_BrO_2_)_2_(C_6_H_6_N_2_O)_2_(H_2_O)_2_] (Hökelek *et al.*, 2009*c*), [Co(C_6_H_6_N_2_O)_2_(H_2_O)_4_](C_8_H_5_O_3_)_2_.2(H_2_O) (Hökelek *et al.*, 2009d), [Co(C_6_H_6_N_2_O)(C_9_H_10_NO_2_)_2_(H_2_O)_2_] (Hökelek *et al.*, 2009e) and [Co(C_8_H_5_O_3_)_2_(C_10_H_14_N_2_O)_2_(H_2_O)_2_] (Sertçelik *et al.*, 2009*b*) have also been determined. In (I), INA ligands are monodentate. The four O atoms (O5, O6, and the symmetry-related atoms, O5', O6') in the equatorial plane around the Ni atom form a slightly distorted square-planar arrangement, while the slightly distorted octahedral coordination is completed by the two pyridine N atoms (N1, N1') of the INA ligands at 2.075 (3) Å from the Ni atom in the axial positions (Fig. 1). The average Ni—O bond length is 2.066 (2) Å. The intramolecular O—H···O hydrogen bonds (Table 1) link the uncoordinated water molecules to the HB anion. The dihedral angle between the planar carboxylate group (O1/O2/C1) and the benzene ring A (C2—C7) is 8.77 (27)°, while that between rings A and B (N1/C8—C12) is 1.53 (11)°.

In the crystal structure, intermolecular O—H···O and N—H···O hydrogen bonds (Table 1) link the molecules into a three-dimensional network, in which they may be effective in the stabilization of the structure. π–π Contacts between the benzene and phenyl rings, *Cg*1···*Cg*2 and *Cg*1···*Cg*2^i^, [symmetry code: (i) -1 + *x*, *y*, *z*, where *Cg*1 and *Cg*2 are centroids of the rings A (C2–C7) and B (N1/C8–C12), respectively] may further stabilize the structure, with centroid-centroid distances of 3.621 (2) and 3.455 (2) Å, respectively.

### Experimental

The title compound was prepared by the reaction of NiSO_4_.6H_2_O (1.314 g, 5 mmol) in H_2_O (100 ml) and INA (1.220 g, 10 mmol) in H_2_O (50 ml) with sodium 3-hydroxybenzoate (1.601 g, 10 mmol) in H_2_O (100 ml). The mixture was filtered and set aside to crystallize at ambient temperature for four weeks, giving blue single crystals.

### Refinement

Atoms H51, H52, H61, H62, H71, H72, H81 and H82 (for H_2_O), H21 and H22 (for NH_2_) and H31 (for OH) were located in difference Fourier map and were refined by applying restraints. C-bound H-atoms were positioned geometrically (C—H = 0.93 Å) and constrained to ride on their parent atoms, with *U*_iso_(H) = 1.2 × *U*_eq_(C).

### Crystal data

**Table:**

[Ni(C_6_H_6_N_2_O)_2_(H_2_O)_4_](C_7_H_5_O_3_)_2_·4H_2_O	*F*(000) = 756
*M**_r_* = 721.29	*D*_x_ = 1.586 Mg m^−^^3^
Monoclinic, *P*2_1_/*n*	Mo *K*α radiation, λ = 0.71073 Å
Hall symbol: -P 2yn	Cell parameters from 7580 reflections
*a* = 6.6884 (3) Å	θ = 2.4–28.4°
*b* = 16.9271 (5) Å	µ = 0.73 mm^−^^1^
*c* = 13.5543 (4) Å	*T* = 100 K
β = 100.186 (3)°	Rod-shaped, blue
*V* = 1510.37 (9) Å^3^	0.46 × 0.33 × 0.18 mm
*Z* = 2	

### Data collection

**Table:**

Bruker Kappa APEXII CCD area-detector diffractometer	3723 independent reflections
Radiation source: fine-focus sealed tube	3365 reflections with *I* > 2σ(*I*)
graphite	*R*_int_ = 0.029
φ and ω scans	θ_max_ = 28.6°, θ_min_ = 1.9°
Absorption correction: multi-scan (*SADABS*; Bruker, 2005)	*h* = −8→8
*T*_min_ = 0.750, *T*_max_ = 0.877	*k* = −22→21
13495 measured reflections	*l* = −17→15

### Refinement

**Table:**

Refinement on *F*^2^	Primary atom site location: structure-invariant direct methods
Least-squares matrix: full	Secondary atom site location: difference Fourier map
*R*[*F*^2^ > 2σ(*F*^2^)] = 0.051	Hydrogen site location: inferred from neighbouring sites
*wR*(*F*^2^) = 0.157	H atoms treated by a mixture of independent and constrained refinement
*S* = 1.26	*w* = 1/[σ^2^(*F*_o_^2^) + (0.0492*P*)^2^ + 5.1781*P*] where *P* = (*F*_o_^2^ + 2*F*_c_^2^)/3
3723 reflections	(Δ/σ)_max_ < 0.001
256 parameters	Δρ_max_ = 0.86 e Å^−^^3^
12 restraints	Δρ_min_ = −0.69 e Å^−^^3^

### Special details

**Table:**

Geometry. All e.s.d.'s (except the e.s.d. in the dihedral angle between two l.s. planes) are estimated using the full covariance matrix. The cell e.s.d.'s are taken into account individually in the estimation of e.s.d.'s in distances, angles and torsion angles; correlations between e.s.d.'s in cell parameters are only used when they are defined by crystal symmetry. An approximate (isotropic) treatment of cell e.s.d.'s is used for estimating e.s.d.'s involving l.s. planes.
Refinement. Refinement of *F*^2^ against ALL reflections. The weighted *R*-factor *wR* and goodness of fit *S* are based on *F*^2^, conventional *R*-factors *R* are based on *F*, with *F* set to zero for negative *F*^2^. The threshold expression of *F*^2^ > σ(*F*^2^) is used only for calculating *R*-factors(gt) *etc*. and is not relevant to the choice of reflections for refinement. *R*-factors based on *F*^2^ are statistically about twice as large as those based on *F*, and *R*- factors based on ALL data will be even larger.

### Fractional atomic coordinates and isotropic or equivalent isotropic displacement parameters (Å^2^)

**Table:**

	*x*	*y*	*z*	*U*_iso_*/*U*_eq_	
Ni1	0.5000	0.0000	0.5000	0.00840 (16)	
O1	−0.0786 (4)	0.04089 (13)	0.29703 (18)	0.0135 (5)	
O2	−0.1778 (4)	0.11616 (14)	0.16276 (17)	0.0145 (5)	
O3	−0.0150 (4)	0.39248 (14)	0.27901 (19)	0.0151 (5)	
H31	−0.011 (8)	0.428 (3)	0.317 (4)	0.027 (13)*	
O4	0.3941 (4)	0.32984 (14)	0.18014 (18)	0.0160 (5)	
O5	0.3225 (4)	0.04752 (14)	0.59662 (18)	0.0128 (5)	
H51	0.372 (9)	0.068 (3)	0.653 (3)	0.051 (17)*	
H52	0.245 (8)	0.014 (3)	0.616 (5)	0.054 (18)*	
O6	0.7696 (4)	0.03386 (14)	0.58654 (19)	0.0148 (5)	
H61	0.803 (7)	0.0796 (16)	0.609 (3)	0.026 (12)*	
H62	0.851 (7)	0.000 (3)	0.618 (4)	0.041 (16)*	
O7	0.0025 (4)	0.51961 (15)	0.40009 (19)	0.0168 (5)	
H71	−0.018 (9)	0.508 (4)	0.453 (2)	0.050*	
H72	−0.101 (6)	0.548 (3)	0.380 (4)	0.047 (17)*	
O8	0.0926 (4)	0.06780 (16)	0.04380 (19)	0.0192 (5)	
H81	0.196 (5)	0.060 (4)	0.077 (4)	0.050*	
H82	0.021 (7)	0.082 (3)	0.085 (3)	0.047 (17)*	
N1	0.4858 (4)	0.10763 (15)	0.4259 (2)	0.0098 (5)	
N2	0.5148 (5)	0.39569 (17)	0.3228 (2)	0.0140 (6)	
H21	0.525 (7)	0.439 (3)	0.296 (3)	0.017 (11)*	
H22	0.559 (8)	0.396 (3)	0.385 (4)	0.030 (13)*	
C1	−0.1092 (5)	0.10742 (18)	0.2549 (2)	0.0112 (6)	
C2	−0.0587 (5)	0.18065 (18)	0.3173 (2)	0.0106 (6)	
C3	−0.0642 (5)	0.25416 (18)	0.2706 (2)	0.0110 (6)	
H3	−0.1005	0.2580	0.2013	0.013*	
C4	−0.0156 (5)	0.32139 (18)	0.3277 (3)	0.0120 (6)	
C5	0.0358 (5)	0.31639 (19)	0.4314 (3)	0.0130 (6)	
H5	0.0684	0.3619	0.4694	0.016*	
C6	0.0386 (5)	0.2433 (2)	0.4783 (3)	0.0137 (6)	
H6	0.0707	0.2399	0.5478	0.016*	
C7	−0.0068 (5)	0.17514 (19)	0.4214 (2)	0.0121 (6)	
H7	−0.0026	0.1261	0.4526	0.015*	
C8	0.4370 (5)	0.11290 (18)	0.3253 (2)	0.0113 (6)	
H8	0.4102	0.0666	0.2884	0.014*	
C9	0.4250 (5)	0.18391 (18)	0.2748 (2)	0.0119 (6)	
H9	0.3897	0.1851	0.2053	0.014*	
C10	0.4660 (5)	0.25372 (18)	0.3287 (2)	0.0106 (6)	
C11	0.5176 (5)	0.24858 (18)	0.4322 (2)	0.0124 (6)	
H11	0.5467	0.2940	0.4706	0.015*	
C12	0.5253 (5)	0.17508 (19)	0.4776 (2)	0.0118 (6)	
H12	0.5594	0.1724	0.5471	0.014*	
C13	0.4552 (5)	0.33050 (18)	0.2717 (2)	0.0115 (6)	

### Atomic displacement parameters (Å^2^)

**Table:**

	*U*^11^	*U*^22^	*U*^33^	*U*^12^	*U*^13^	*U*^23^
Ni1	0.0102 (3)	0.0075 (3)	0.0072 (3)	−0.00025 (19)	0.00071 (19)	0.00018 (19)
O1	0.0146 (11)	0.0103 (10)	0.0151 (12)	0.0006 (8)	0.0015 (9)	0.0001 (8)
O2	0.0171 (12)	0.0151 (11)	0.0105 (11)	−0.0004 (9)	0.0007 (9)	−0.0006 (9)
O3	0.0218 (13)	0.0101 (11)	0.0132 (12)	0.0002 (9)	0.0022 (10)	0.0006 (9)
O4	0.0246 (13)	0.0122 (11)	0.0107 (11)	0.0019 (9)	0.0016 (10)	0.0017 (8)
O5	0.0145 (12)	0.0136 (11)	0.0113 (11)	−0.0003 (8)	0.0049 (9)	−0.0006 (9)
O6	0.0155 (12)	0.0098 (11)	0.0162 (12)	0.0000 (9)	−0.0052 (9)	−0.0013 (9)
O7	0.0199 (13)	0.0139 (11)	0.0155 (12)	0.0031 (9)	0.0004 (10)	0.0000 (9)
O8	0.0202 (13)	0.0214 (13)	0.0173 (13)	0.0031 (10)	0.0064 (10)	0.0001 (10)
N1	0.0084 (12)	0.0117 (12)	0.0096 (12)	0.0005 (9)	0.0025 (10)	0.0005 (9)
N2	0.0199 (15)	0.0109 (13)	0.0105 (14)	−0.0009 (10)	0.0007 (11)	0.0027 (10)
C1	0.0072 (13)	0.0127 (14)	0.0142 (15)	0.0003 (10)	0.0030 (11)	−0.0011 (11)
C2	0.0079 (14)	0.0115 (14)	0.0126 (15)	0.0008 (10)	0.0022 (11)	−0.0012 (11)
C3	0.0095 (14)	0.0147 (14)	0.0088 (14)	0.0014 (11)	0.0015 (11)	0.0008 (11)
C4	0.0094 (14)	0.0108 (14)	0.0160 (16)	0.0009 (11)	0.0027 (12)	0.0020 (12)
C5	0.0117 (14)	0.0129 (14)	0.0141 (16)	0.0003 (11)	0.0019 (12)	−0.0030 (11)
C6	0.0133 (15)	0.0172 (15)	0.0101 (15)	0.0010 (11)	0.0010 (12)	0.0001 (12)
C7	0.0129 (15)	0.0116 (14)	0.0124 (15)	0.0017 (11)	0.0034 (12)	0.0020 (11)
C8	0.0107 (14)	0.0113 (14)	0.0118 (15)	−0.0002 (11)	0.0019 (11)	−0.0009 (11)
C9	0.0120 (14)	0.0132 (14)	0.0105 (15)	0.0002 (11)	0.0016 (12)	0.0006 (11)
C10	0.0092 (14)	0.0102 (13)	0.0125 (15)	0.0011 (10)	0.0024 (11)	0.0018 (11)
C11	0.0143 (15)	0.0100 (14)	0.0129 (15)	0.0003 (11)	0.0026 (12)	−0.0011 (11)
C12	0.0107 (14)	0.0135 (14)	0.0115 (15)	0.0007 (11)	0.0028 (11)	−0.0003 (11)
C13	0.0100 (14)	0.0119 (14)	0.0133 (15)	0.0022 (11)	0.0038 (12)	0.0020 (11)

### Geometric parameters (Å, °)

**Table:**

Ni1—O5	2.079 (2)	N2—H21	0.83 (5)
Ni1—O5^i^	2.079 (2)	N2—H22	0.84 (5)
Ni1—O6	2.052 (2)	C2—C1	1.505 (4)
Ni1—O6^i^	2.052 (2)	C2—C3	1.394 (4)
Ni1—N1	2.075 (3)	C2—C7	1.395 (4)
Ni1—N1^i^	2.075 (3)	C3—H3	0.9300
O1—C1	1.263 (4)	C4—C3	1.383 (4)
O2—C1	1.260 (4)	C5—C4	1.389 (5)
O3—C4	1.373 (4)	C5—C6	1.390 (5)
O3—H31	0.79 (5)	C5—H5	0.9300
O4—C13	1.236 (4)	C6—H6	0.9300
O5—H51	0.85 (2)	C7—C6	1.391 (5)
O5—H52	0.85 (2)	C7—H7	0.9300
O6—H61	0.85 (2)	C8—H8	0.9300
O6—H62	0.85 (2)	C9—C8	1.378 (4)
O7—H71	0.784 (18)	C9—C10	1.391 (4)
O7—H72	0.85 (2)	C9—H9	0.9300
O8—H81	0.769 (18)	C10—C11	1.386 (5)
O8—H82	0.83 (2)	C10—C13	1.507 (4)
N1—C8	1.347 (4)	C11—H11	0.9300
N1—C12	1.341 (4)	C12—C11	1.385 (4)
N2—C13	1.326 (4)	C12—H12	0.9300
			
O5—Ni1—O5^i^	180.00 (9)	C7—C2—C1	120.3 (3)
O6—Ni1—O5	94.23 (10)	C2—C3—H3	120.1
O6^i^—Ni1—O5	85.77 (10)	C4—C3—C2	119.7 (3)
O6—Ni1—O5^i^	85.77 (10)	C4—C3—H3	120.1
O6^i^—Ni1—O5^i^	94.23 (10)	O3—C4—C3	118.2 (3)
O6^i^—Ni1—O6	180.0	O3—C4—C5	121.2 (3)
O6—Ni1—N1	89.53 (10)	C3—C4—C5	120.5 (3)
O6^i^—Ni1—N1	90.47 (10)	C4—C5—C6	119.9 (3)
O6—Ni1—N1^i^	90.47 (10)	C4—C5—H5	120.1
O6^i^—Ni1—N1^i^	89.53 (10)	C6—C5—H5	120.1
N1—Ni1—O5	89.02 (10)	C5—C6—C7	120.1 (3)
N1^i^—Ni1—O5	90.98 (10)	C5—C6—H6	120.0
N1—Ni1—O5^i^	90.98 (10)	C7—C6—H6	120.0
N1^i^—Ni1—O5^i^	89.02 (10)	C2—C7—H7	120.1
N1^i^—Ni1—N1	180.0	C6—C7—C2	119.8 (3)
C4—O3—H31	111 (4)	C6—C7—H7	120.1
Ni1—O5—H51	123 (4)	N1—C8—C9	122.8 (3)
Ni1—O5—H52	113 (4)	N1—C8—H8	118.6
H52—O5—H51	99 (6)	C9—C8—H8	118.6
Ni1—O6—H61	128 (3)	C8—C9—C10	119.4 (3)
Ni1—O6—H62	121 (4)	C8—C9—H9	120.3
H61—O6—H62	110 (5)	C10—C9—H9	120.3
H72—O7—H71	100 (4)	C9—C10—C13	118.5 (3)
H81—O8—H82	103 (4)	C11—C10—C9	117.9 (3)
C8—N1—Ni1	122.0 (2)	C11—C10—C13	123.6 (3)
C12—N1—Ni1	120.4 (2)	C10—C11—H11	120.4
C12—N1—C8	117.6 (3)	C12—C11—C10	119.3 (3)
C13—N2—H21	123 (3)	C12—C11—H11	120.4
C13—N2—H22	123 (3)	N1—C12—C11	122.9 (3)
H21—N2—H22	113 (4)	N1—C12—H12	118.5
O1—C1—C2	118.5 (3)	C11—C12—H12	118.5
O2—C1—O1	123.7 (3)	O4—C13—N2	123.2 (3)
O2—C1—C2	117.8 (3)	O4—C13—C10	119.0 (3)
C3—C2—C1	119.6 (3)	N2—C13—C10	117.9 (3)
C3—C2—C7	120.1 (3)		
			
O5—Ni1—N1—C8	−131.1 (3)	C1—C2—C7—C6	−179.7 (3)
O5^i^—Ni1—N1—C8	48.9 (3)	C3—C2—C7—C6	0.2 (5)
O5—Ni1—N1—C12	48.7 (2)	O3—C4—C3—C2	177.5 (3)
O5^i^—Ni1—N1—C12	−131.3 (2)	C5—C4—C3—C2	−0.9 (5)
O6—Ni1—N1—C8	134.6 (3)	C6—C5—C4—O3	−178.4 (3)
O6^i^—Ni1—N1—C8	−45.4 (3)	C6—C5—C4—C3	0.0 (5)
O6—Ni1—N1—C12	−45.5 (2)	C4—C5—C6—C7	1.1 (5)
O6^i^—Ni1—N1—C12	134.5 (2)	C2—C7—C6—C5	−1.2 (5)
Ni1—N1—C8—C9	179.4 (2)	C10—C9—C8—N1	0.5 (5)
C12—N1—C8—C9	−0.5 (5)	C8—C9—C10—C11	−0.1 (5)
Ni1—N1—C12—C11	−179.8 (2)	C8—C9—C10—C13	179.0 (3)
C8—N1—C12—C11	0.1 (5)	C9—C10—C11—C12	−0.3 (5)
C3—C2—C1—O1	171.2 (3)	C13—C10—C11—C12	−179.4 (3)
C3—C2—C1—O2	−8.0 (4)	C9—C10—C13—O4	6.0 (5)
C7—C2—C1—O1	−8.9 (5)	C9—C10—C13—N2	−173.2 (3)
C7—C2—C1—O2	171.9 (3)	C11—C10—C13—O4	−175.0 (3)
C1—C2—C3—C4	−179.3 (3)	C11—C10—C13—N2	5.8 (5)
C7—C2—C3—C4	0.8 (5)	N1—C12—C11—C10	0.3 (5)

Symmetry codes: (i) −*x*+1, −*y*, −*z*+1.

### Hydrogen-bond geometry (Å, °)

**Table:**

*D*—H···*A*	*D*—H	H···*A*	*D*···*A*	*D*—H···*A*
N2—H21···O1^ii^	0.83 (5)	2.20 (5)	3.018 (4)	169 (4)
N2—H22···O8^iii^	0.84 (5)	2.21 (5)	3.012 (4)	159 (5)
O3—H31···O7	0.79 (5)	1.91 (5)	2.696 (4)	175 (5)
O5—H51···O3^iii^	0.85 (4)	1.87 (4)	2.716 (3)	177 (5)
O5—H52···O1^iv^	0.84 (5)	1.99 (6)	2.795 (4)	160 (6)
O6—H61···O4^iii^	0.85 (3)	1.86 (3)	2.693 (3)	169 (4)
O6—H62···O1^i^	0.85 (5)	1.87 (5)	2.685 (4)	159 (5)
O7—H71···O7^v^	0.78 (4)	2.03 (3)	2.795 (4)	167 (6)
O7—H72···O2^vi^	0.85 (5)	1.88 (5)	2.731 (4)	177 (5)
O8—H81···O7^vii^	0.77 (4)	2.10 (4)	2.802 (4)	152 (6)
O8—H82···O2	0.83 (4)	1.93 (5)	2.752 (4)	171 (4)

Symmetry codes: (ii) −*x*+1/2, *y*+1/2, −*z*+1/2; (iii) *x*+1/2, −*y*+1/2, *z*+1/2; (iv) −*x*, −*y*, −*z*+1; (i) −*x*+1, −*y*, −*z*+1; (v) −*x*, −*y*+1, −*z*+1; (vi) −*x*−1/2, *y*+1/2, −*z*+1/2; (vii) −*x*+1/2, *y*−1/2, −*z*+1/2.
